# Long-term trends in injury-related mortality among children and adolescents: a 32-year study in a central urban district of Shanghai, 1993–2024

**DOI:** 10.3389/fpubh.2026.1794295

**Published:** 2026-04-10

**Authors:** Yun Zhang, Jiaxin Huang, Shuna Gao, Yi Tang, Yijun Wang, Yan Yu, Zhiyi Ling, Weiyi Li

**Affiliations:** 1 Department of Comprehensive Operations and Emergency Response, Shanghai Huangpu District Center for Disease Control and Prevention (Shanghai Huangpu District Health Supervision Institute), Shanghai, China; 2 Department for Prevention of Tumor and Injury, Shanghai Huangpu District Center for Disease Control and Prevention (Shanghai Huangpu District Health Supervision Institute), Shanghai, China; 3 Department of Injury Prevention and Control, Institute of Chronic Non-communicable Diseases and Injury Prevention and Control, Shanghai Municipal Center for Disease Control and Prevention (Shanghai Academy of Preventive Medicine), Shanghai, China

**Keywords:** adolescents, children, injury surveillance, injury-related mortality, suicide, traffic injuries

## Abstract

**Background:**

This study aims to examine trends in injury-related mortality among children and adolescents aged 0–19 years in a central urban district of Shanghai from 1993 to 2024, as well as its epidemiological characteristics.

**Methods:**

This study is a retrospective study. From 1993 to 2024, data were obtained from the Shanghai Cause-of-Death Surveillance System. Death records with injury as the underlying cause that were categorized under the International Statistical Classification of Diseases and Related Health Problems (ICD) codes V01-Y89 (external causes of injury and poisoning) were gathered.

**Results:**

For children and adolescents aged from 0 to 19, the standardized injury-related mortality rate was 5.21 per 100,000, and 8.87% of all-cause mortality. The top two causes of injury-related mortality were suicide (1.15 per 100,000) and traffic injuries (1.02 per 100,000). Leading causes of injury-related mortality varied by age group: traffic injuries were the primary cause of injury-related death in the 5–9 age group; accidental poisoning and suicide were most common for the 10–14 age group, traffic injuries and suicide were the most prevalent causes in the 15–19 age group. The crude mortality rate of the 0–4 year old age group has shown a fluctuating low-level trend since 2003. The peak of accidental falls and drowning deaths in the 0–4 age group occurred in 2003. In the 5–19 years group, road traffic injuries exhibited intermittent peaks, while suicide showed a persistent upward trend. From 1993 to 2004, accidental poisoning and traffic injuries were the primary causes, while suicide and traffic injuries ranked first and second between 2005 and 2014. From 2015 to 2024, suicide surpassed traffic injuries as the primary cause of death. During the study period, the overall standardized injury-related mortality among children and adolescents aged 0–19 years in Huangpu District showed fluctuations (APC = −0.875, *p* = 0.420). However, significant declining trends were observed for traffic injuries, drowning, accidental falls, and accidental poisoning (*p* < 0.05).

**Conclusion:**

Different types of injuries exhibit gender and age differences, suggesting the need for targeted interventions according to the developmental trends and characteristics of children and adolescents.

## 1 Introduction

Injuries poses significant worldwide public health challenge, not only severely compromising population health, but also is the number one cause of death and disability, particularly among infants (1, 2). According to the latest data from United Nations International Children’s Emergency Fund (UNICEF), over 1,600 children and adolescents under the age of 19 die everyday from injuries globally, with road traffic injuries being the number one cause of mortality in this age group (3). Furthermore, World Health Organization (WHO) published a report that injuries “are considered the greatest threat to children’s lives and health in the 21st century” (4). The health loss from injuries is accompanied by enormous socioeconomic expense, through both direct medical costs and indirect losses in productivity, resulting in lasting consequences for families and society (5). However, injures are preventable. Though the application of scientifically designed, evidence-based interventions, the incidence of injuries can be reduced (6).

As one of the most populous countries in the world, China has experienced rapid demographic transition, urbanization, and motorization expansion over the past four decades. These transformations have deeply changed injury patterns and population distribution (7, 8). While injuries are prevalent throughout the nation, their epidemiological patterns differentiated across the region. Economic development levels, transportation environments, population density, residential structures, and social resources significantly influence injury types and risk exposure (8). These environmental factors are characteristic of Huangpu- a high density central district in Shanghai. As a large urban district, Huangpu developed a unique environmental feature shaped by the rapid changing economic and demographic trends. The urban environment is featured as the high population density, complex transportation networks, high concentration of high-rise buildings, and heavily utilization rate of public facilities. The environmental characteristics differ from suburb or urban environment, the injured risk pattern and exposure factors of local children and adolescents are expected to possess unique complexity and specificity.

Current epidemiology research of injury-related mortality among children and adolescents in China mainly focusing on national or provincial level (8–10), however there are very few studies analyzing long-term trends in high density urban central district of city. Understanding various injury patterns and long-term trends is important to establish sustained, evidence-based injury prevention strategies. Hence, this study aims to systematically assess the long-term trends and the epidemiological characteristics of injury-related mortality among children and adolescents aged 0–19 in a central urban district of Shanghai from 1993 to 2024. We hope our findings helps to provide scientific evidence for developing injury prevention strategies.

## 2 Methods

### 2.1 Study design, study population, setting and outcome

This study was designed as a retrospective study. The study population were all registered residents aged 0–19 in a central urban district of Shanghai (Huangpu District). Outcomes of this study were considered as recorded deaths due to injury-related mortality.

Data for the study population were obtained from the Public Security Bureau. Injury-related mortality data were obtained from the cause-of-death registration records in the Shanghai Cause of Death Registration System from 1993 to 2024. The administrative boundary adjustments and data processing for Huangpu District, Shanghai are as follow: Huangpu District underwent two major administrative boundary adjustments during the study period. In 2000, the former Nanshi District was dissolved, with parts incorporated into Huangpu District. In 2011, Luwan District was merged with Huangpu District to form the new Huangpu District. To ensure comparability of the 32-year time series data have a consistent geographic scope, all historical data underwent standardized processing. The core principle is mapping all annual data to the current administrative boundary of Huangpu District, using the standard code “310,101” as the unique identifier. Hence, this study’s dataset covers data from Nanshi District, Luwan District, and Huangpu District for the years 1993 onwards.

### 2.2 Cause-of-death classification

Causes of death were coded according to the International Classification of Diseases (ICD). From 1993 to 2002, the Ninth Revision (ICD-9) (11) was used, in which injury-related internal cause diagnosis codes ranged from 800 to 999 (including fractures, dislocations, burns, open wounds, and spinal cord injuries), and external cause codes ranged from E800-E999 (including falls, traffic injuries, drowning, and other injury mechanisms). Since 2003, cause-of-death coding has followed the Tenth Revision (ICD-10) (12). Injury-related deaths were identified using external cause codes V01-Y89 (V00-Y99), corresponding to injuries and poisoning, with internal cause diagnosis codes classified under S00-T98.

In this study, injury categories included asphyxia (W75-W84), electric shock (W85-W99), accidental poisoning (X40-X49), suicide (X60-X84), homicide (X85-Y09), and fire-related injuries (X00-X09), while remaining injury mechanisms were grouped as “Other” (12, 13) (Table 1).

**Table 1:** Shanghai ICD bridge table.

Categories	Cause group	ICD9 codes	ICD10 codes
Transport accidents	Motor vehicle/non-motor vehicle traffic injuries	E800-E848	V01-V99
	Among them: Pedestrians	E810-E825(0.7)	V01-V09
	Among them: Cyclists	E800-E807(0.3), E810-E825(0.6)	V10-V19
	Among them: Motorcycles	E810-E825(0.2/0.3)	V20-V29
Accidental falls	Accidental Falls	E880-E888	W00-W19
Drowning	Drowning	E910	W65-W74
Accidental poisoning	Accidental Poisoning	E850-E869	X40-X49
Fire burn	Fire Burn	E890-E899, E924	X00-X19
mechanical asphyxia	mechanical asphyxia	E911-E913	W75-W84
Self-harm	Self-harm	E950-E959	X60-X84
Intentional injuries	Intentional injuries	E960-E969	X85-Y09
Uncertain intention	Uncertain intention	E980-E989	Y10-Y34
Others	Others	E900-E949	W20-W64, X20-X59, Y35-Y89

### 2.3 Quality control

Injury-related mortality cases were identified through the primary cause of death listed as injury on the “Medical Death Certificate for Residents,” “Death Inference Certificate for Residents,” and “Death Confirmation Certificate for Residents” issued by medical institutions at all levels in Huangpu District, Shanghai, as well as the “Death Confirmation Certificate for Residents” issued by public security departments. Cases were reported through the Shanghai Cause of Death Registration System. The Huangpu District Center for Disease Control and Prevention (CDC) conducted individual reviews of all death information of each case, followed by the re-examination by the Shanghai CDC. All data was quality controlled by disease prevention and control professionals. Infant deaths in Huangpu District would review by clinical, public health and other specialist panels, with causes of death clearly established. This ensured a 100% death registration rate and the proportion of unexplained deaths below 5%. Moreover, all mortality codes for children under 5 years of age would underwent individual quality control by the Shanghai CDC. All cases that did not show causes of injuries were investigated at the community level through household inspections to confirm data originality, rationality, and completeness. During the study period, the Huangpu District, the registered resident deaths reporting rate was 100.00%, the reviewed rate of deaths through time was 100.00%, cause-of-death unknown rate was <5.00%. Cause-of-death surveillance data has been continuously complete and maintained high quality.

### 2.4 Primary analytical indicators

Key analytical indicators in this study include crude death rate, standardized death rate, composition ratio, Potential Years of Life Lost (PYLL), Average Years of Life Lost (AYLL), Potential Years of Life Lost Rate (PYLLR) and Annual percent change (APC). The Years of Life Lost formulas are as follow: PYLL = ∑[(L-Xi) × Di], AYLL = PYLL/d, PYLLR = (PYLL/N) × 1,000‰. Here, *L* = 70 represents the upper age threshold; Xi is defined as the median age in the i-th age group; Di represents the number of deaths in the i-th age group; N represents the total actual population; d represents the number of deaths in specific cause in same interval. APC was used to express trends of mortality rate overtime. Age-standardized injury mortality as the dependent variable and the year as the independent variable, the linear regression model was fitted to analyze the APC.

### 2.5 Statistical analysis

Data were organized and analyzed using SPSS 21.0 and Excel 2021 software. Joinpoint Regression Program (Version 5.0.4, National Cancer Institute, Calverton, United States) was used to calculate APC and perform trend analysis of mortality rates, with a significance level of *p* = 0.05. To ensure the model adequately captures potential complex trends and avoid overfitting, we set the max joinpoints for six and grid step for 1 year. To eliminate the influence of population structure and ensure comparability, mortality rates were standardized using the population from the 7th National Population Census released in 2020.

### 2.6 Ethics statement

Ethical approvals were obtained from Shanghai Huangpu District Center for Disease Control and Prevention (Shanghai Huangpu District Health Supervision Institute), Shanghai, China. Project number: HPEC202601.

## 3 Results

### 3.1 Overview of population characteristics and injury mortality

From 1993 to 2024, children and adolescents aged 0–19 years accounted for 15.08% (4,518,298/29,954,645) of the total population in Huangpu District, Shanghai. Deaths in this age group contributed 0.98% (2,773/2,833,760) to all-cause mortality, with injury-related mortality contributed 8.87% (246/2,773) of all-cause mortality. Annual average all-cause mortality in the study area was 6271.74 per 100,000. All-cause mortality and injury-related mortality among study population was 61.37 per 100,000 and 5.44 per 100,000, respectively. A total of 246 injury-related deaths were recorded among people aged 0–19, including 141 males and 105 females. Annual crude mortality rate and annual standardized mortality rate was 5.44 per 100,000 and 5.21 per 100,000, respectively. Males have higher crude and standardized injury-related mortality rates and a higher proportion of injury-related mortality (6.09, 5.59 per 100,000 and 9.95%) compared to females (4.76, 4.88 per 100,000 and 7.74%) (Table 2).

**Table 2:** Injury-related mortality among children and adolescents aged 0–19 in Huangpu District, Shanghai, 1993–2024.

Year	Male			Female			Total population		
	Crude death rate per 100,000 population	Standardized death rate per 100,000 population	All-cause mortality rate (%)	Crude death rate per 100,000 population	Standardized death rate per 100,000 population	All-cause mortality rate (%)	Crude death rate per 100,000 population	Standardized death rate per 100,000 population	All-cause mortality rate(%)
1993	7.30	7.40	6.33	3.85	4.12	3.11	5.62	5.82	4.70
1994	6.73	6.85	5.06	5.51	5.69	3.63	6.14	6.31	4.31
1995	10.81	12.08	6.60	5.69	5.95	3.43	8.32	9.01	5.05
1996	7.92	8.79	5.21	7.45	6.88	4.27	7.69	7.82	4.71
1997	9.82	9.71	18.18	3.41	4.63	5.48	6.68	7.17	11.51
1998	5.07	5.42	10.53	3.49	4.12	5.63	4.29	4.78	7.81
1999	6.12	7.31	10.14	5.41	6.79	8.57	5.77	7.03	9.35
2000	9.27	9.49	16.39	6.82	8.19	11.86	8.07	8.83	14.17
2001	5.88	5.88	9.68	6.17	4.97	12.00	6.02	5.45	10.71
2002	4.66	5.52	14.81	2.42	1.12	7.41	3.56	3.36	11.11
2003	2.59	1.88	9.09	6.73	11.91	22.73	4.62	6.75	15.91
2004	1.39	0.62	6.67	1.44	0.63	5.26	1.41	0.63	5.88
2005	6.13	6.81	33.33	0.00	0.00	0.00	3.12	3.48	16.67
2006	6.42	4.16	16.00	3.32	6.15	22.22	4.90	2.83	17.65
2007	5.13	3.75	17.65	8.84	8.29	33.33	6.95	5.94	25.00
2008	0.00	0.00	0.00	7.74	6.71	25.00	3.79	3.30	11.76
2009	6.00	6.34	13.64	4.20	4.91	25.00	5.12	5.67	16.67
2010	8.38	7.16	23.53	2.19	3.43	9.09	5.36	5.33	17.86
2011	6.39	4.94	16.67	4.47	4.28	20.00	5.45	4.67	17.86
2012	2.12	1.59	5.56	2.23	1.73	7.69	2.17	1.70	6.45
2013	2.08	1.48	7.69	2.18	2.81	7.69	2.13	2.16	7.69
2014	6.18	5.64	15.79	0.00	0.00	0.00	3.18	2.92	11.11
2015	0.00	0.00	0.00	2.19	1.56	8.33	1.06	0.74	3.33
2016	0.00	0.00	0.00	4.40	4.61	15.38	2.13	2.25	6.90
2017	2.07	2.79	6.25	8.79	7.81	33.33	5.32	5.21	17.86
2018	10.41	10.15	38.46	4.44	4.14	28.57	7.52	7.26	35.00
2019	8.46	9.76	25.00	0.00	0.00	0.00	4.37	4.96	19.05
2020	2.16	2.54	16.67	9.22	6.47	57.14	5.57	5.83	38.46
2021	6.69	6.93	50.00	4.77	4.71	33.33	5.76	5.81	41.67
2022	6.92	6.72	42.86	12.40	12.57	55.56	9.56	9.51	50.00
2023	7.13	7.02	42.86	5.12	6.35	33.33	6.16	6.61	38.46
2024	9.64	10.28	28.57	5.21	4.60	50.00	7.51	7.60	33.33
Annual Average	6.09	5.59	9.95	4.76	4.88	7.74	5.44	5.21	8.87

### 3.2 Injury mortality types by population group

Among children and adolescents aged 0–19 years, males have a higher crude injury-related mortality rate (6.09 per 100,000) compared to females (4.76 per 100,000). Furthermore, injury-related mortality accounted for 9.95% of the total male deaths, compared to 7.74% for females. The top three causes of injury-related mortality were suicide (1.15 per 100,000), traffic injuries (1.02 per 100,000) and accidental poisoning (0.71 per 100,000). An analysis was conducted on the age groups of 0–4 years old and 5–19 years old, the crude mortality rate of the 0–4 year old age group showed a fluctuating low level trend after 2003. The crude mortality rate of accidental falls and drowning in this age group reached its peak in 2003, and there have been no incidents of accidental falls among children aged 0–4 since 2003, and no drowning incidents since 2015. The mortality rate of injuries in the age group of 5–19 fluctuates greatly. In unintentional injuries, road traffic accidents are the main type, showing fluctuating peak characteristics, and there were no road traffic injuries or deaths throughout 2022–2024; In intentional injury, suicide shows a continuous fluctuating upward trend.

By age group, infants under 1 year of age had the highest injury-related mortality rate (18.15 per 100,000), 50% (13/26) of them were due to accidental injuries and other harmful effects. Among children aged 1–4, injury-related mortality rate was 3.05 per 100,000, with traffic injuries being the leading cause of injury-related mortality among both males (1.13 per 100,000) and females (0.60 per 100,000). Among children aged 5–9, the injury-related mortality rate was 3.55 per 100,000, with traffic injuries being the leading cause of injury death for both males (0.71 per 100,000) and females (1.50 per 100,000). Among children aged 10–14, the injury-related mortality trends exhibited vibrations. For males, the leading cause was traffic injuries (1.41 per 100,000), followed by suicide (0.94 per 100,000). For females, the leading cause was accidental poisoning (1.33 per 100,000), followed by suicide (0.99 per 100,000). Among adolescents aged 15–19, suicide emerged as the primary causes of injury-related mortality for both genders. For males, suicide ranked first (3.08 per 100,000), followed by traffic injuries (1.90 per 100,000). For females, suicide also ranked first (2.87 per 100,000), followed by accidental poisoning (0.91 per 100,000) (Table 3).

**Table 3:** Primary causes of injury-related mortality among children and adolescents in Huangpu District, Shanghai, by gender and age group, 1993–2024.

Types of injury-related mortality	Under 1 year		1–4 years		5–9 years		10–14 years		15–19 years		0–19 years	
	Death cases	Crude rate*	Death cases	Crude rate*	Death cases	Crude rate*	Death cases	Crude rate*	Death cases	Crude rate*	Death cases	Crude rate*
Males												
Accidental falls	1	1.35	2	0.57	3	0.53	4	0.63	4	0.59	14	0.61
Traffic injuries	0	0.00	4	1.13	4	0.71	9	1.41	13	1.90	30	1.30
Suicide	0	0.00	0	0.00	0	0.00	6	0.94	21	3.08	27	1.17
Accidental poisoning	0	0.00	1	0.28	2	0.35	4	0.63	10	1.46	17	0.73
Drowning	1	1.35	4	1.13	4	0.71	2	0.31	10	1.46	21	0.91
Others	10	13.49	3	0.85	6	1.06	6	0.94	7	1.03	32	1.38
Total	12	16.19	14	3.96	19	3.37	31	4.85	65	9.52	141	6.09
Females												
Accidental falls	0	0.00	2	0.60	4	0.75	3	0.50	2	0.30	11	0.50
Traffic injuries	0	0.00	2	0.60	7	1.31	2	0.33	5	0.75	16	0.73
Suicide	0	0.00	0	0.00	0	0.00	6	0.99	19	2.87	25	1.13
Accidental poisoning	0	0.00	0	0.00	1	0.19	8	1.33	6	0.91	15	0.68
Drowning	1	1.45	2	0.60	3	0.56	0	0.00	0	0.00	6	0.27
Others	13	18.82	1	0.30	5	0.94	8	1.33	5	0.75	32	1.45
Total	14	20.26	7	2.09	20	3.74	27	4.47	37	5.59	105	4.76
0verall												
Accidental falls	1	0.70	4	0.58	7	0.64	7	0.56	6	0.45	25	0.55
Traffic injuries	0	0.00	6	0.87	11	1.00	11	0.89	18	1.34	46	1.02
Suicide	0	0.00	0	0.00	0	0.00	12	0.97	40	2.97	52	1.15
Accidental poisoning	0	0.00	1	0.15	3	0.27	12	0.97	16	1.19	32	0.71
Drowning	2	1.40	6	0.87	7	0.64	2	0.16	10	0.74	27	0.60
Others	23	16.06	4	0.58	11	1.00	14	1.13	12	0.89	63	1.39
Total	26	18.15	21	3.05	39	3.55	58	4.67	102	7.58	246	5.44

*Per 100,000 population.

**Figure 1:**
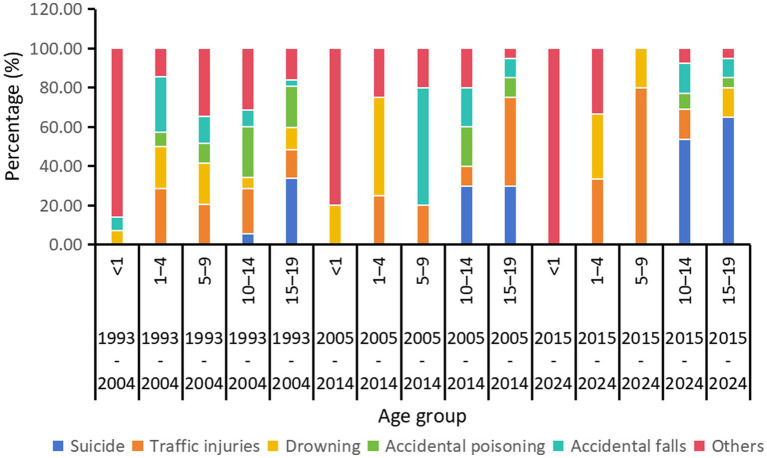
Cause-specific composition of injury mortality by age group and time period, Huangpu District, Shanghai, 1993–2024.

Analysis of injury mortality among children and adolescents in Huangpu District from 1993 to 2024 reveals distinct temporal trends and age-specific patterns. Figure 1 presents the proportional distribution of injury causes by year (1993–2004, 2005–2014, and 2015–2024). Suicide emerged as the predominant cause among adolescents (15–19 year olds) in recent years. In contrast, while traffic injuries still remains a significant factor, it showed a declining trend. For accidental poisoning, most cases concentrated between 1993 and 2004, particularly among those aged 10–19. After 2004, its contribution declined substantially. For accidental falls, the injury-related mortality showed a transient peak in 2005–2014, particularly among children aged 5–14. However, the mortality declined in recent period (2015–2024).

### 3.3 Life years lost analysis

From 1993 to 2024, the cumulative PYLL for children and adolescents aged 0–19 in Huangpu District, Shanghai, was 14,341.5 person-years, with AYLL at 58.3 years and PYLLR at 3.17‰. AYLL for male (57.83 years) was lower than female (58.92 years), while cumulative PYLL and PYLLR for male (8,154.5 person-years and 3.52‰) were higher than female (6,187 person-years, 2.81‰). The top three causes of injury for PYLL and PYLLR in both male and female were suicide, accidental poisoning, and traffic injuries, while the top three causes for AYLL in both male and female were drowning, accidental falls, and traffic injuries (Table 4).

**Table 4:** Analysis of life years lost due to injury-related deaths among residents of Huangpu District, Shanghai, 1993–2024.

Types of injury-related mortality	Males			Females			Total		
	PYLL (person-years)	AYLL (years)	PYLLR (‰)	PYLL (person-years)	AYLL (years)	PYLLR (‰)	PYLL (person-years)	AYLL (years)	PYLLR (‰)
Accidental falls	831	59.36	0.36	661.50	60.14	0.30	1492.50	59.70	0.33
Traffic injuries	2729.5	58.07	1.18	1011.50	59.50	0.46	3741.00	58.45	0.83
Suicide	1447.5	53.61	0.63	1342.50	53.70	0.61	2790.00	53.65	0.62
Accidental poisoning	947	55.71	0.41	837.50	55.83	0.38	1784.50	55.77	0.39
Drowning	1618.5	59.94	0.36	391.00	65.17	0.18	1618.50	59.94	0.36
Others	1983.5	61.98	0.86	1943.00	62.68	0.88	3926.50	62.33	0.87
Total	8154.50	57.83	3.52	6187.00	58.92	2.81	14341.50	58.30	3.17

### 3.4 Temporal trends in major injury types

**Figure 2:**
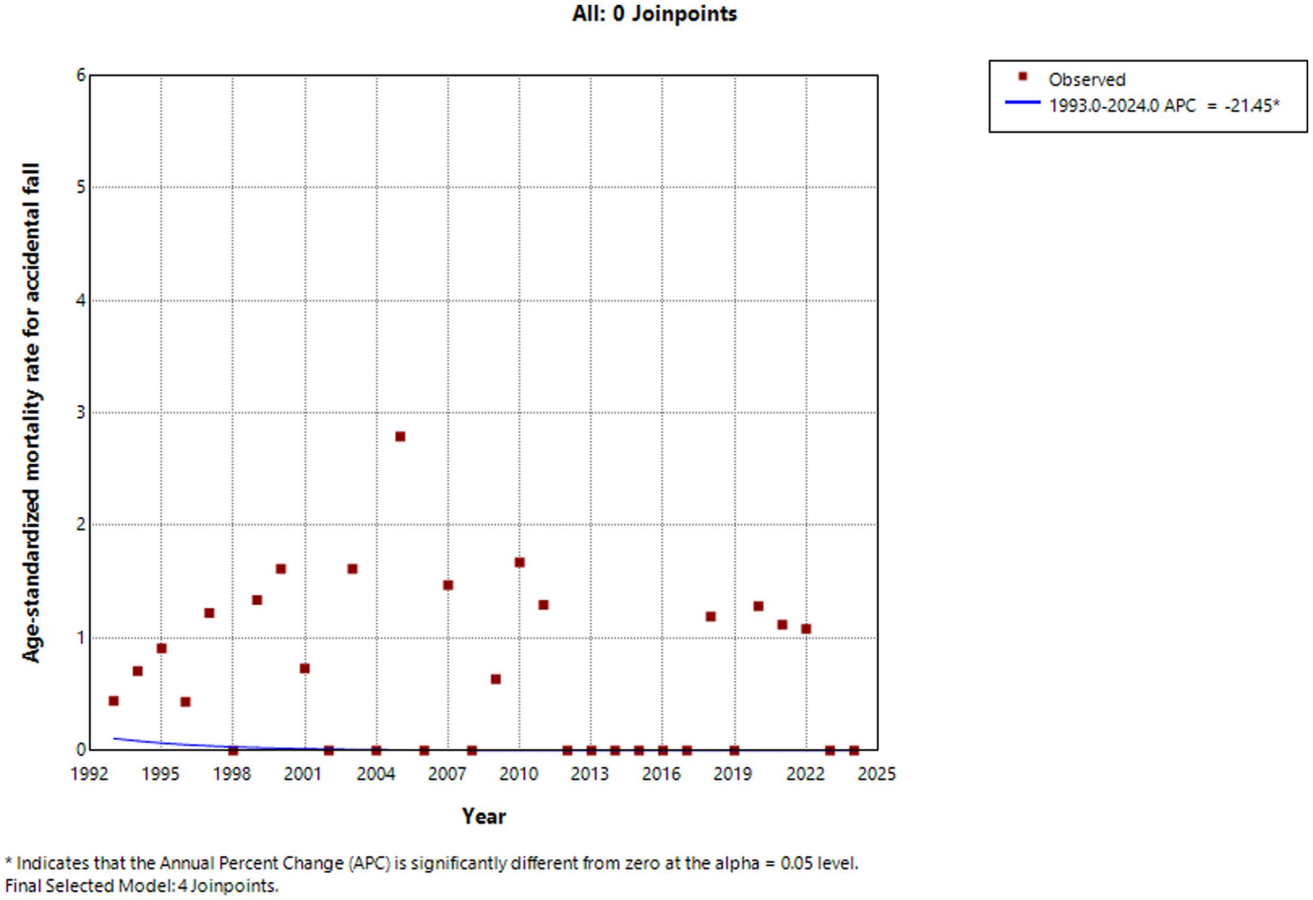
Accidental falls standardized mortality rate (per 100,000 people) trends among children and adolescents aged 0–19 years in Huangpu District, Shanghai.

**Figure 3:**
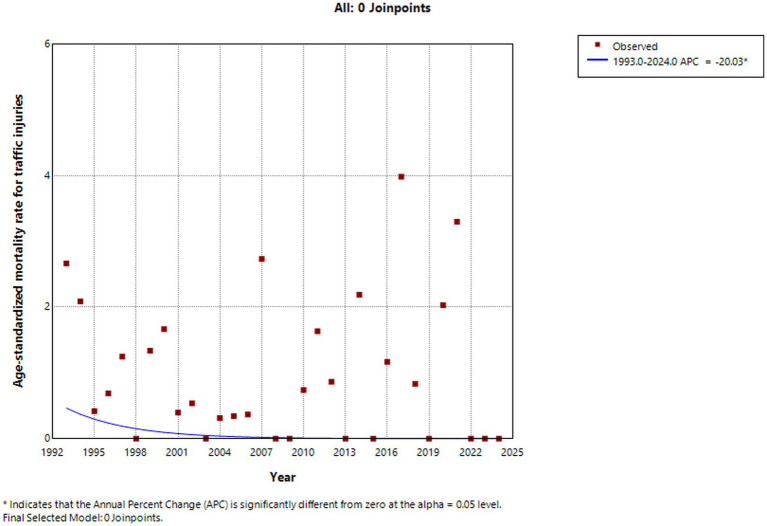
Traffic injuries standardized mortality rate (per 100,000 people) trends among children and adolescents aged 0–19 years in Huangpu District, Shanghai.

**Figure 4:**
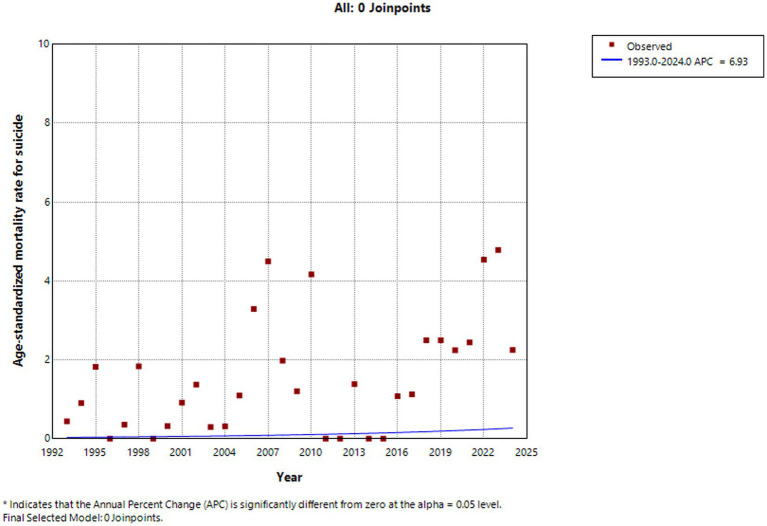
Suicide standardized mortality rate (per 100,000 people) trends among children and adolescents aged 0–19 years in Huangpu District, Shanghai.

**Figure 5:**
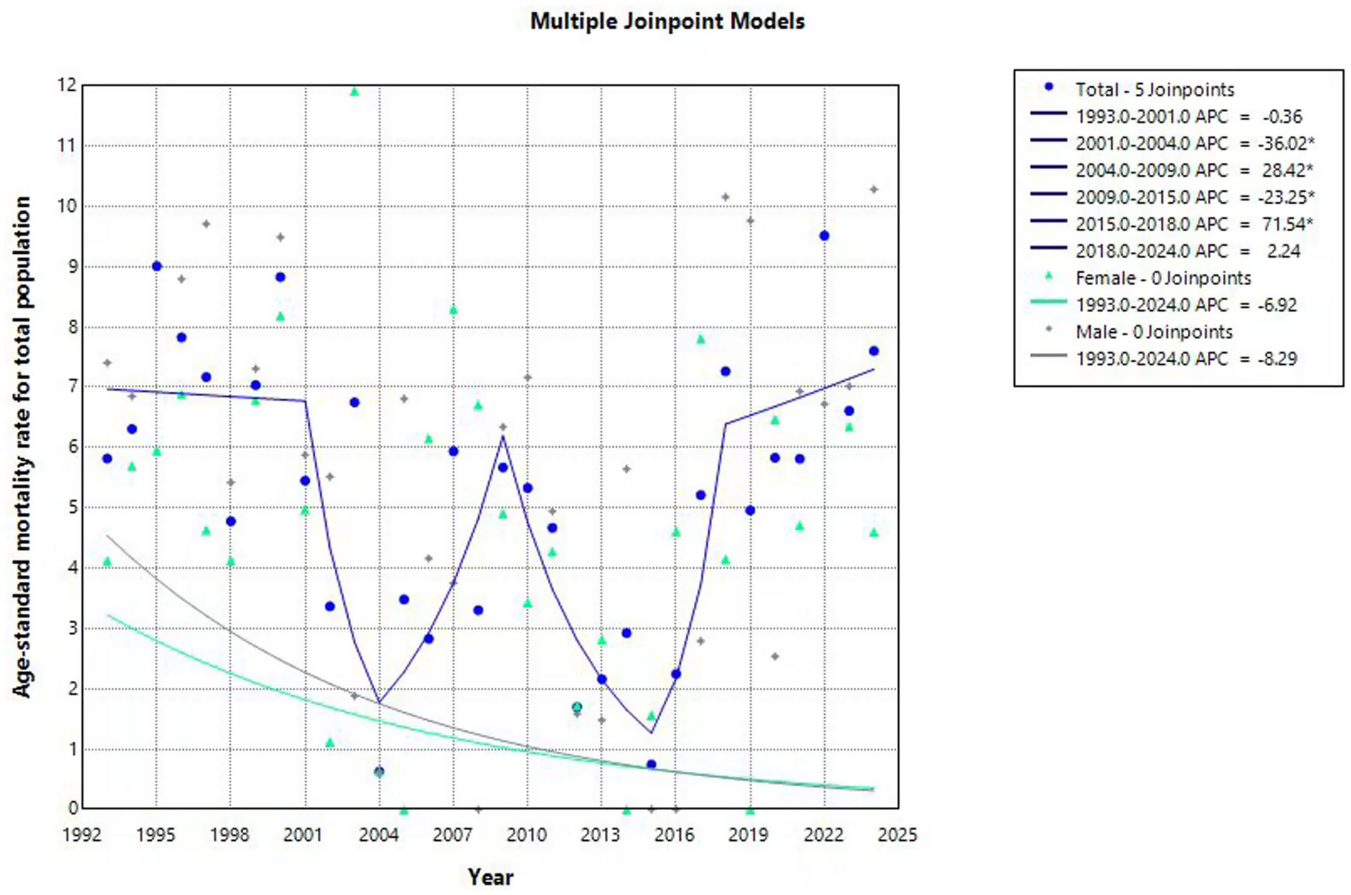
Total population age-standardized mortality rate (per 100,000 people) trends among children and adolescents aged 0–19 years in Huangpu District, Shanghai.

From 1993 to 2024, the standardized injury-related mortality rate among children and adolescents aged 0–19 years in Huangpu District showed fluctuations over time, with no statistically significant trend detected across the entire study period (APC = −0.875, *p* = 0.420). Decreased from 1993 to 2004, increased between 2004 and 2009, and then decreased again from 2010 to 2015. This was followed by a sharp increase between 2015 and 2018, after which the rate showed a modest upward trend from 2018 to 2024. The standardized suicide-related mortality rate exhibited a fluctuating pattern over the 32-year period, with no statistically significant upward trend (APC = 6.932%, *p* = 0.530), but there was a decline in suicide-related mortality was observed from 2023 to 2024. In contrast, statistically significant downward trends were observed for several specific injury types. Traffic injuries (APC = −20.033%, *p* = 0.034), accidental falls (APC = −21.45%, *p* = 0.020), drowning (APC = −22.807%, *p* = 0.049), and accidental poisoning (APC = −35.224%, *p* < 0.001) all showed significant declines over the study period. Injury-related mortality rates among both males (APC = −8.289%, *p* = 0.349) and females (APC = −6.919%, *p* = 0.361) shown no statistically significant changes (Figures 2–5).

## 4 Discussion

Over the 32-year study period (1993–2024), the overall standardized injury mortality rate among children and adolescents aged 0–19 years in Huangpu District showed fluctuating but no statistically significant trend (APC = −0.875, *p* = 0.420). However, declining trends were observed for traffic injuries, drowning, accidental falls, and accidental poisoning (*p* < 0.05). In recent years, suicide and traffic injuries have emerged as the two leading causes of injury-related mortality, with suicide showing a fluctuating trend.

Our study suggests that during the study period, the annual average injury-related mortality rate for children and adolescents aged 0–19 in Huangpu District was 5.44 per 100,000. This result was comparable to studies conducted in high-income Asia Pacific area, western Europe area (14) and Beijing (15). These differences may suggest a positive correlation between regional economic development and the allocation of public health resources, environmental safety, and the sophistication of prevention systems, which plays an essential role in furthering injury prevention for children and adolescents (16). Despite this, the substantial PYLL (14,341.5 person-years) and AYLL (58.3 years) underscore the persistent burden of preventable deaths.

The suicide mortality rate among children aged 0–19 from 1993 to 2024 is 1.15 per 100,000. Although lower than rates reported in many high-income settings, rising concerns about youth suicide have been documented globally. In the United States, suicide rates among persons aged 15–19 increased by 76% between 2007 and 2017, from 6.7 to 11.8 per 100,000 (17). In Singapore, adolescent suicide rates nearly doubled from 5.35 per 100,000 in 2019 to 9.14 per 100,000 in 2021 among those aged 10–19 years (18). In South Korea, the adolescent female suicide rates increased by 10.91% annually between 2015 and 2023 (19). In recent years, suicide emerged as the dominant cause among adolescents (15–19 years). As the core urban center of Shanghai, Huangpu district has a unique socio-environmental landscape that contributes to this trend through three primary mechanisms. First, the high concentration of well-known educational resources drives intense academic competition, creating long-term pressure. The continual pursuit of academic excellence is strongly associated with increased adolescent anxiety, depression, and suicidal ideation (20, 21). Second, the high living costs and the socioeconomic disparities in this premium urban district can foster feelings of relative deprivation and social comparison. Evidence from similar affluent urban settings suggests that such economic pressures contribute to psychological distress and family conflict, both of which are risk factors for youth suicide (18). Third, the urban lifestyle syndrome, characterized by social isolation despite high population density, weakened community bonds, and excessive digital engagement, would further compromises mental health. A Japanese study indicates that the combination of internet addiction and chronic sleep deprivation significantly increases adolescent psychological vulnerability (22).

A notable finding of this study is the significant decline in suicide mortality among adolescents aged 15–19 years in Huangpu District during the 2023–2024 period. Three primary factors may help explain this positive trend. First, the “lagged effect” of long-term mental health policies is likely beginning to manifest. Initiatives such as the China National Program for Child Development (2021–2030) and the Huangpu District Child-Friendly City Construction Action Plan have institutionalized school-based mental health services and crisis intervention mechanisms. Second, improvements in mental health service accessibility such as the expansion of school counseling services, the establishment of youth-friendly mental health hotlines, and the increased mental health awareness campaigns have lowered barriers to help-seeking. Third, the post-pandemic recovery (2020–2022) facilitated a return to structured routines and in-person social connections, which serve as essential buffers against psychological distress. To better understand the causes of the three positive changes, future research incorporating service utilization data and qualitative interviews is needed. International evidence suggests several essential practices for sustaining this progress (17–19). These include early identification through screening tools like HEADSSS (Home, Education, Activities, Drugs, Sexuality, Suicide/Depression, Safety) in primary care, strengthening school-community-mental health service linkages, regulating access to medications and lethal methods, adhering to responsible media reporting guidelines, and fostering multi-sectoral collaborations to address academic stress and bullying.

The significant downward trends of traffic-related mortality coincides with the implementation of several key national and local prevention policies. To improve the transportation environment around schools, Shanghai has implemented cross-departmental management strategies. These include the “one school, one policy” traffic management plan tailored to schools and kindergartens, the “shared helmet” program to promote student cycling safety, and the 2017 mandate for child safety seats (23). Beyond infrastructure and equipment, community-based teams comprising public security, social workers, and counselors, have further supported student safety and well-being through education and mental health initiatives. Collectively, these multifaceted, collaborative interventions have contributed substantially to the observed decline in traffic-related mortality among children and adolescents (24, 25).

Significant declining trends were also observed for traditional unintentional injuries, specifically drowning, accidental falls, and accidental poisoning. These injuries are closely linked to environmental hazards and inadequate adult supervision (26). Evidence indicates that enhancing environmental safety, strengthening water safety education, and regulating hazardous substances are effective strategies for reducing these incidents (27, 28). The favorable trends in this study is likely reflect the cumulative impact of multi-level policies and interventions. At the national level, the China National Program for Child Development (2021–2030) has prioritized the reduction of drowning, falls, and poisoning (29). Locally, the Huangpu District Child-Friendly City Action Plan strengthens injury surveillance and safety management within schools and residential communities (30). Ultimately, the integration of sustained household and community level interventions, and enhanced parental supervision (31), have contributed to these consistent declines.

Throughout the study period, males experienced a higher risk of injury-related mortality and a greater burden of years of life lost. This finding aligns with pervious study (14) and may because of their distinct behavioral patterns. Compared to girls, boys tend to engage in more frequent risk-taking behaviors, experience longer periods of unsupervised outdoor activity, and participate more frequently in high-risk recreational activities (32). These behavioral patterns directly increase their exposure to high-risk environments for traffic incidents, drowning, and serious falls (33). Furthermore, the reasons of the suicidal ideation seems to differ by gender. Among boys frequently associated with physical fighting and prior injuries, while among girls associated with social vulnerabilities—such as bullying exposure and a lack of close friendships (34). Consequently, there is an urgent need for early, contextually tailored interventions that address both male risk-taking and female social vulnerabilities. Given the rising prevalence of school and cyberbullying, innovative multi-sectoral approaches that combine community, parental, and mobile health interventions are essential for effective early prevention.

In this study, infants under 1 year accounted for 50% of the cases were classified as “accidental injuries and other harmful effects.” National data indicate that suffocation is the leading cause of infant injury death in China, representing 62.3% of cases (9). This suggests that most unspecified deaths in our study were likely sleep-related suffocations. According to the American Academy of Pediatrics, key modifiable risk factors include prone sleeping (OR = 2.3–13.1), soft bedding (5-fold risk), and bed-sharing (OR = 2.88, 95% CI: 1.99–4.18), with infants under 4 months of age being at particularly high risk due to immature motor skills and limited ability to escape potential threats (35). To mitigate these risks, a safe sleep environment is essential. Supine positioning, the use of a firm sleep surface, room-sharing without bed-sharing, the avoidance of soft bedding and overheating, and the need to continue targeted prevention efforts with a focus on safe sleep practices and caregiver education was recommended.

Findings of this study highlight both progress and challenges in child injury prevention. To sustain this progress, future efforts should include strengthening enforcement of existing traffic safety and product safety regulations, and expanding mental health infrastructure, include training more child psychiatrists and embedding school counselors within school settings. Addressing emerging risks through internet safety education and cyberbullying prevention is equally vital, as it is the promotion of responsible media reporting to mitigate the risk of suicide contagion. Furthermore, enhancing surveillance systems with greater granularity will be essential to track emerging threats and evaluate the long-term impact of the policy.

This study has several limitations. First, external environmental factors contributing to various injury types (e.g., injury-causing objects, environments) were not investigated in depth. Future study should incorporate injury-specific cause-of-death surveys to better understand these factors. Second, regional injury incidence rates and case numbers remains unclear, with injury patterns and disease burden were investigated from a mortality perspective. Third, while the COVID-19 pandemic did not affect the completeness of death reporting, it may have influenced the occurrence patterns of certain injury types. For example, reduced mobility during lockdowns may have contributed to the decline in traffic injuries. Hence, future studies with longer post-pandemic follow-up are necessary to further elucidate these potential effects. Finally, with our models identify trends, the application of more advanced statistical modeling could provide deeper insights into the complex underlying temporal dynamics of child injury mortality.

In summary, although injury-related mortality among children and adolescents aged 0–19 in Huangpu District, Shanghai, shows an overall downward trend, challenges posed by suicide and traffic injuries need continued attention. Conducting targeted interventions according to the developmental trends and features of children and adolescents has been recommended. These efforts should focus on the popularization of knowledge and skills in emergency rescue, as well as improvement in prevention and response capacities on multiple levels, which include the family, schools, and society.

## Data Availability

The original contributions presented in the study are included in the article/supplementary material, further inquiries can be directed to the corresponding author.
